# Ultra sub-wavelength surface plasmon confinement using air-gap, sub-wavelength ring resonator arrays

**DOI:** 10.1038/srep22305

**Published:** 2016-02-29

**Authors:** Jaehak Lee, Sangkeun Sung, Jun-Hyuk Choi, Seok Chan Eom, N. Asger Mortensen, Jung H. Shin

**Affiliations:** 1 Korea Advanced Institute of Science and Technology, Department of Physics, 373-1 Guseong-dong, Yuseong-Gu, Daejeon, South Korea; 2 Korea Institute of Machinery & Materials, 156 Gajeongbuk-Ro, Yuseong-Gu, Daejeon, South Korea; 3 Technical University of Denmark, Department of Photonics Engineering, DK-2800 Kongens Lyngby, Denmark; 4 Korea Advanced Institute of Science and Technology, Graduate School of Nanoscience and Technology, 373-1 Guseong-dong, Yuseong-Gu, Daejeon, South Korea

## Abstract

Arrays of sub-wavelength, sub-10 nm air-gap plasmonic ring resonators are fabricated using nanoimprinting. In near infra-red (NIR) range, the resonator supports a single dipole mode which is excited and identified via simple normal illumination and explored through transmission measurements. By controlling both lateral and vertical confinement via a metal edge, the mode volume is successfully reduced down to 1.3 × 10^−5^ λ_0_^3^. The advantage of such mode confinement is demonstrated by applying the resonators biosensing. Using bovine serum albumin (BSA) molecules, a dramatic enhancement of surface sensitivity up to 69 nm/nm is achieved as the modal height approaches the thickness of the adsorbed molecule layers.

The propagation of electromagnetic waves can be manipulated at the nanoscale using metallic nanostructures, via coupling with surface plasmons^1^,^2^,^3^,^4^,^5^,^6^,^7^,^8^,^9^. In case of noble metals, their high plasma frequency and relatively low Ohmic resistivity result in a very high absolute value of Re(ϵ) in the infra-red. Thus, when two such noble metal parts separated by a narrow gap are used for coupling with electromagnetic waves, the requirement for continuity of the perpendicular D-field component results in a huge enhancement of the electric field in the gap. Such a strong enhancement of the E-field in a small volume is highly advantageous not only for many diverse applications such as sensors^10^,^11^,^12^,^13^, lasers^14^,^15^,^16^,^17^, and color displays^18^,^19^,^20^,^21^ but also for fundamental control of optical phenomena^22^,^23^. Consequently, such gap structures, also called metal-insulator-metal (MIM) structures^24^ or gap plasmons^25^, have been the subject of intense research activities^26^,^27^,^28^,^29^,^30^,^31^,^32^,^33^,^34^,^35^.

A widely used method to fabricate such a narrow gap structure is to form a vertical gap by etching through a metal film using either focused ion-beam (FIB) or electron-beam lithography^11^,^12^,^13^,^36^,^37^. Precise drawings of arbitrary shapes are possible, but at the cost of long process time and high fabrication cost. Furthermore, such methods are difficult to scale up to a large-scale fabrication. An alternative method is to define a gap via sequential deposition of metal, dielectric, and metal thin films^15^,^21^,^22^,^26^,^29^,^38^. In this case, smooth, nm-thin gaps can be fabricated with relative ease, as they are defined simply by the deposited dielectric layer. However, the physical presence of the dielectric layer prevents access to the high E-field from outside (e.g. by chemical analytes), significantly limiting the range of possible applications. Furthermore, as the gap confines the E-field in one direction only (i.e., the field component perpendicular to the gap), the optical field can spread extensively parallel to the metal layers.

Indeed, for an effective confinement of the optical field, careful design of the metal structure beyond mere gap formation is necessary. One structure that was shown to be effective for tight field confinement is a T-shaped gap in which a sharp tip is placed above a metal layer^39^. Unfortunately, fully controlled three-dimensional fabrication of such a T-shaped gap is difficult to achieve using either vertical or horizontal gap structures. Recently, we have reported on fabricating a ring resonator with such a junction structure using a directional deposition of metal onto a slot-disk. In this way the T-shaped gap is formed along the circumference of the disk between the sidewall of the top disk and the top surface of the bottom disk. A gap as narrow as 4 nm, with a cross-sectional mode area of 7 × 10^−5^ λ_0_^2^ could be fabricated using photolithography, and the possibility of application for a biosensor was demonstrated^35^. However, the resonators were rather large, with a diameter of 3.5 μm, leading to large overall mode volume. Moreover, the excitation and detection of resonances required a tapered fiber coupling, which does not lend to easy, cost-effective applications.

In this paper, we report on fabricating a large-area array of sub-wavelength sized disk resonators, with T-shaped metal air-gap of sub 10-nm size controlled via deposition method^27^,^31^,^40^. The T-shaped gap confines the optical mode laterally and vertically, while the sub-wavelength size of the resonator localizes the optical mode longitudinally. Consequently, the resonator supports only a single dipole mode near the wavelength of 1500 nm with a mode volume of only 1.3 × 10^−5^ λ_0_^3^ that can be excited efficiently by simple top illumination. Furthermore, the high density of uniform resonators in the array, made possible by nanoimprinting, results in very high transmission efficiency of 1.4~13% such that the plasmon resonance can easily be explored in simple transmission measurements. Finally, we demonstrate the advantage of such resonator arrays for biosensor applications. For surface coverage of the BSA molecules, the gap mode showed surface sensitivity up to 69 nm/nm as the modal height approaches the thickness of the adsorbed BSA layers.

## Results

### Formation of the 5-nm air gap

**Figure 1:**
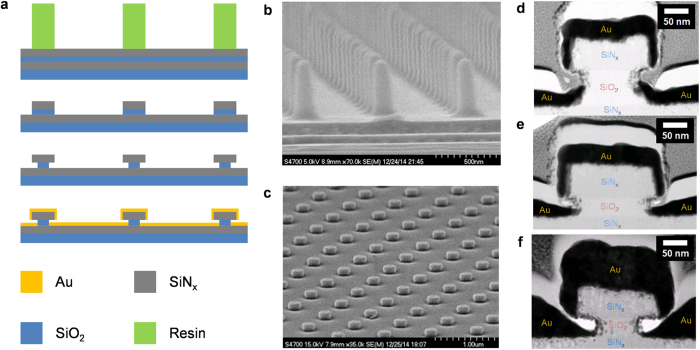
Fabrication of the plasmonic air-gap ring resonator arrays. (**a**) Schematics of the fabrication process in cross-sectional view. (**b**) SEM image of imprinted pillar arrays on the multilayers. (**c**) SEM image of the fabricated resonator arrays. (**d–f**) TEM cross-section image of the resonator fabricated with different gap sizes of (**d**) 40 nm, (**e**) 15 nm and (**f**) 5 nm. The materials are indicated on each regions. The other regions of the TEM images, are protection films coated just before FIB milling.

The fabrication process for the resonator arrays are summarized in Fig. 1a. First, a SiN_x_/SiO_2_/SiN_x_ multilayer film was deposited on a quartz substrate. Afterward, a hexagonal array of polymer pillars with 200 nm diameter and 800 nm pitch was formed via nanoimprinting method^41^. Using the pillars as etch masks, SiN_x_ disks were formed in the top SiN_x_ layer. The SiO_2_ layer was then etched selectively using buffered oxide etchant to undercut the SiN disks, thus forming a slot. Finally, an Au layer with a Cr adhesion layer was deposited to form MIM structure at the edges of the SiN_x_ slots (See method section for more details). A scanning electron microscope (SEM) image of the imprinted pillars are shown in Fig. 1b, and an SEM image of the final resonator array is shown in Fig. 1c. A large array of uniformly sized, button-shaped resonators on the basal metal layer can be seen. More details about the structure can be seen in Fig. 1d–f, which shows the cross-sectional transmission electron microscope (TEM) images of the resonators. We find that the presence of the undercut slot effectively turns the top disk into a shadow mask during the metal deposition such that after the deposition, a well-defined gap is formed between the metal cap on the top SiN_x_ disk and the basal metal layer on the bottom SiN_x_ layer. Moreover, despite of the shadowing by the top disk, the basal gold layer extends into the gap below the top SiN disk, while the bottom edge of the sidewalls of the top gold cap forms a rounded tip opposite to the top surface of the basal Au bottom ring due to the self-shadowing effect. Furthermore, as the gap is defined by deposition, the root-mean-square (RMS) roughness of the surfaces is 0.8 nm only^35^. The result is a narrow, T-shaped gap that confines the E-field both vertically and laterally. The degree of lateral and vertical confinement can be controlled with nanometer precision by controlling the SiO_2_ spacer layer thickness and the metal thickness, and the size of the disk determines the longitudinal confinement via the resonance condition of the whispering gallery mode (WGM). The final gap size, defined to be the shortest distance between the bottom edge of the top gold gap and the basal gold layer, could be controlled down to 5 nm. An analysis of TEM images of a series of 5-nm gap resonators fabricated under identical conditions indicated a standard deviation of 1.6 nm in the gap sizes as well.

### Calculated plasmon modes in the air-gap resonator

**Figure 2:**
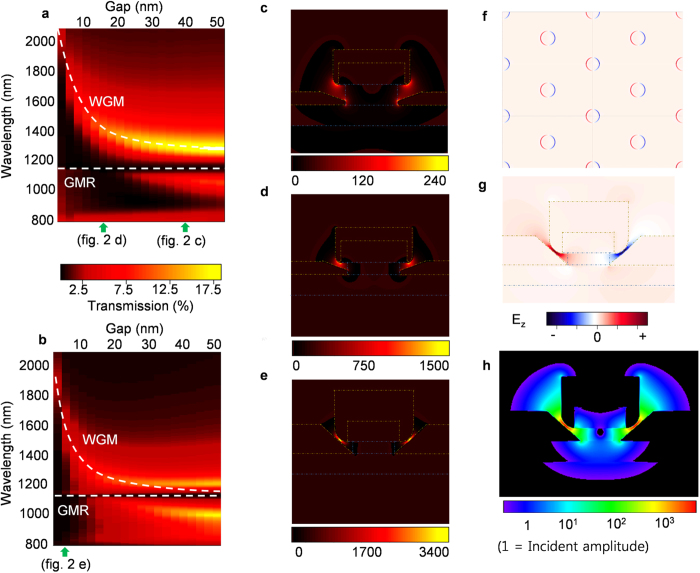
FDTD Simulations. (**a,b**) Calculated transmission spectra upon varying gap sizes and metal thicknesses of (**a**) 45 nm and (**b**) 90 nm. The resonance wavelengths for each modes are indicated as white dashed lines. (**c–e**) |E|^2^ distributions of the resonance modes calculated based on structures reconstructed from the TEM images. (**c**) Cross-sectional view of the mode of the 40 nm gap resonator (Fig. 1d), (**d**) mode of 15 nm gap resonator (Fig. 1e), (**e**) mode of 5 nm gap resonator (Fig. 1f). (**f,g**) Normal components of the E-field (E_z_), (**f**) shows the top view of the mode of the 5 nm gap resonator in the plane of the air-gap, while (**g**) shows the cross-sectional view of same mode. (**h**) Cross-sectional Log|E|^2^ distribution of the mode in the 5 nm gap resonator.

Figure 2a,b show transmission spectra with varying gap sizes (2.5 nm to 50 nm) and two different metal thickness (45 nm and 90 nm), as calculated by finite-difference time-domain (FDTD) simulations. We find that in the near infra-red rage of λ > 800 nm, there exist only a single transmission peak that blueshifts with increasing gap size and metal thickness^34^,^35^,^39^. The transmission peak appears to split into two as the gap size increase beyond 15 nm. However, this is due to a transmission minimum located at λ = 1,142 nm, as shall be shown later. Figure 2c–h show the calculated optical modes at wavelengths corresponding to transmission peak, as indicated in Fig. 2a,b. Actual structures obtained from TEM images (as shown in Fig. 1d–f) were used in calculations. We find that the transmission peaks correspond to excitation of dipole-like plasmonic WGM, confined tightly within the T-shaped gap along the circumference of the disk. Consequently, the overall mode volume is very small – as small as 1.3 × 10^−5^ λ_0_^3^ for the resonator shown in Fig. 2e–h. We also note that such concentration of the mode in the air gap also indicates that the mode is an anti-symmetric mode, where the charges on the basal metal layer and the charges on the tip of the sidewall edge have opposite signs. The absence of other resonance modes in our spectra is ascribed to the resonator’s small size and highly non-symmetric shape that were shown to favor excitation of dipole-like modes^35^. The transmission can be quite high – as high as 18% of the incident light for the 40 nm gap resonator, even though the gap size is much smaller than the wavelength of the incident light, and in any case, the gaps are invisible in the Au film when viewed vertically due to the overlap of the top Au disks and the basal Au layer. This is because the gap-plasmonic WGM acts as a channel which, at resonance, selectively funnels the incident light through the gap and out into the far field. This can be seen in Fig. 2h that shows the calculated electric field intensity of the resonance modes in logarithm scale. The Supplementary Movie S1 (a) and (b) also show propagations of resonant and non-resonant light, respectively, normally incident on a 5-nm gap resonator. At resonance, the incident wave is funneled through the gap so that a large fraction of the incident light is transmitted. Off resonance, the incident wave is nearly completely reflected, with very little transmission through the resonator.

### Measurement of resonant transmission through the gap

**Figure 3:**
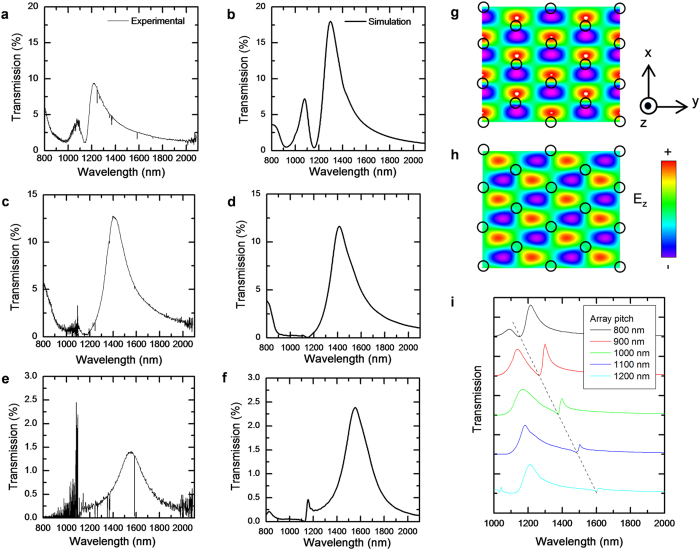
Transmission spectra. (**a–f**) Transmission spectra of the ring resonator arrays, (**a**) experimental and (**b**). FDTD simulated transmission spectra of the 40 nm gap resonator (Fig. 1d), (**c,d**) same for 15 nm gap resonator (Fig. 1e), (**e,f**) same for the 5 nm gap resonator (Fig. 1f). (**g,h**) Top view of normal components of the E-field (E_z_) in the plane of the SiN_x_ layer underneath the Au film when 1,141 nm light, coincident with the dip, is incident normally upon the array of the resonators of 40 nm gap, with polarizations of E_x_ (**g**) and E_y_ (**h**). Black circles indicate the positions of the resonators. (**i**) Calculated transmission spectra of the ring resonators arrays when the pitch is varied from 800 nm to 1,200 nm.

The dipole-like gap-plasmonic WGMs also enable their efficient excitation by vertical illumination, and their observation in a simple transmission measurement. Figure 3a–f show the calculated and measured transmission spectra for the resonator arrays shown in Fig. 1d–f. The agreement between measured and observed spectra is excellent, with an experimental transmission of 13% obtained from the resonator with 15 nm gap. The transmission is reduced to 1.4% for the resonator with 5 nm gap, but even in this case, the signal to noise ratio for the gap plamonic WGM is as high as 100. For all resonators, a dip in the transmission at 1142 nm is clearly evident, as discussed above. The position of this dip is independent of the gap size, and corresponds to excitation of guided mode resonance (GMR)^42^ due to the periodic boundary conditions of the array structures. This is confirmed by Fig. 3g,h, which show that at 1142 nm, the E-field is concentrated in spaces between the disks, with exactly one wavelength of the propagating wave fitting into the space between the nearest neighbors in the SiN_x_ layer underneath the basal Au film. Indeed, as Fig. 3i shows, the position of the dip is calculated to be proportional to the pitch.

#### Surface sensitivity

**Figure 4:**
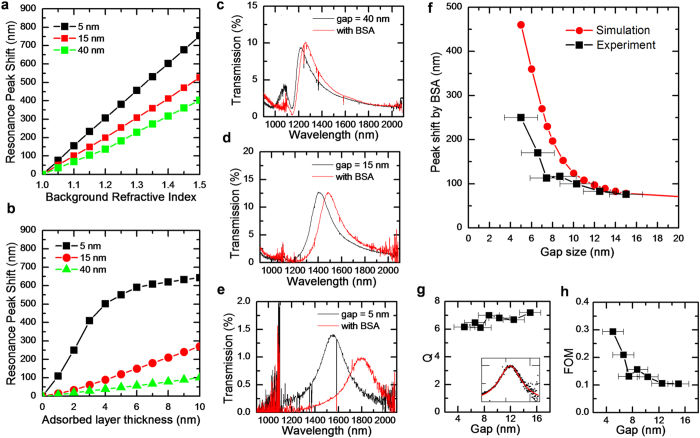
Biosensing. (**a**) Calculated bulk sensitivities of the resonators with 5 nm, 15 nm and 40 nm gaps. (**b**) Calculated resonance peak shifts due to surface layer adsorption of BSA. (**c–e**) Transmission spectra of the (**c**) 40-nm gap, (**d**) 15-nm gap and (**e**) 5-nm gap resonators before (black) and after (red) surface saturation with 10% BSA solution. (**f**) Calculated (red) and experimental (black) dependence of the peak shift on the gap size, after surface saturation with 10% BSA solution. (**g**) Experimental Q factor acquired from the Loretzian fitting (inset) and (**h**) FOM. All measurements and calculations were conducted against air-background and error bars represents the standard deviations of gap sizes measured from TEM images.

In order to confirm the results of simulations and measurements, and to reveal the advantage of the resonator structures, we demonstrate sensitivity for surface change of the resonator arrays. We first investigate numerically the sensitivity of the resonator structure. As shown in Fig. 4a, when the structures are fully emerged in an analyte, the bulk sensitivity is estimated to be 800~1,500 nm/RIU, quite high among the resonator-based sensors but modest when compared to existing Kretschmann SPR systems^43^. However, we note that bulk sensitivity is simply proportional to the extension of the optical mode into the analyte, and does not fully reflect the advantage of the molecular range optical confinement provided by gap plasmon resonators. This is demonstrated in Fig. 4b, which shows the calculated values of surface sensitivity, defined to be the resonance peak shift per unit thickness of the adsorbed analyte material^44^,^45^,^46^,^47^, assuming a refractive index of BSA^48^,^49^, as a model analyte. We find that, as expected, narrower gaps give higher sensitivity. At the same time, it is also clear that such high sensitivity is obtained at the cost of dynamic range, as the peak shift quickly saturates for the narrowest gap resonators.

The calculated surface sensitivity is confirmed experimentally by observing the shift of the resonance peak upon covering the resonator array with a monolayer of BSA, as saturation coverage of Au films with BSA is reported to result in formation of a monolayer of 3.5 nm thickness and 1.5 refractive index^49^,^50^,^51^. BSA layer was formed by dipping the array structure in a 10% BSA solution for 3 hrs at room temperature. The uniformity of the BSA layer was confirmed by the near independence of the changes in the transmission spectra upon the location of the beam spot (data not shown). Figure 4c–e shows the measured transmission spectra for resonator arrays shown in Figs 1, 2, 3 after BSA coverage. For the resonators with large gaps, an accurate estimate of the peak shift is difficult due to the presence of the transmission dip at 1142 nm, as discussed above. However, for resonators with estimated gap sizes of 15 nm and 5 nm, a clear shift of 75 nm and 250 nm, corresponding to surface sensitivity of 21 nm/nm and 69 nm/nm, respectively, can be observed. We note that it is possible to shrink the gap even further to 3 nm, which would give an even higher peak shift; however, experimental verification of such a large shift was not possible due to limitations of our measurement setup (See Supplementary Figure S2 online). The dependence of the peak shift upon the gap sizes is summarized in Fig. 4f. As shown in Fig. 4f, the experimental value agrees very well with calculated value when the gap size is large (15 nm). In case of resonators with smaller gaps (5 nm ~12 nm), the experimentally observed peak shifts are smaller than the theoretically predicted values, which we attribute to difficulty of the BSA molecules in fully penetrating the gap that is smaller than the size of BSA molecule (4 nm × 4 nm × 14 nm)^52^.

#### Q factor and Figure of merit (FOM)

For biosensing and many other applications, another important resonator parameter is the Q factor of the resonator. Figure 4g shows the measured and calculated Q factors of resonators with different gap sizes, as obtained from Lorentzian fittings on the experimental transmission spectra. As the gap size decreases from 15 nm to 5 nm with concomitant decrease of mode volume from 8.2 × 10^−5^ λ_0_^3^ to 1.3 × 10^−5^ λ_0_^3^ and increase in the surface sensitivity from 21 nm/nm to 69 nm/nm, the Q factor remains nearly constant, decreasing from 7.2 to 6.2 only. Consequently, the FOM of the resonator-based biosesnors, which we define to be FOM = (Sensitivity/FWHM)^53^ is also highly enhanced upon reduction of the gap sizes, as shown in Fig. 4h.

## Discussions

The demonstrated mode volume 1.3 × 10^−5^ λ_0_^3^ is one of the smallest values ever reported for WGM resonators^38^. Indeed, the mode volume is smaller than even that of a bow-tie antenna^16^, and clearly demonstrates the advantage of the present structure in providing such a high degree of 3-dimensional confinement. In fact, the observed 250 nm of resonance shift indicates that 30% of the mode energy is utilized to sense the monolayer of the BSA molecules, with about 60 molecules contributing to the observed peak shift. In other words, a single BSA molecule contributes, on the average, a peak shift of 4.2 nm. Therefore, given a peak position resolution of 1.2 nm (data not shown), we believe that even a single molecule in the gap can be detected, which will be demonstrated in a subsequent report. In addition, the observed surface sensitivity is among the highest values ever reported for surface sensitivity from a resonator structure, being 59, 12, and 2~3 times higher than those reported for conventional evanescent-field sensors^46^, dielectric-based slot resonators^45^, and Au/Ag nanoparticle based LSPR sensors^48^, respectively, when normalized by the operation wavelength. We note that a similarly high sensitivity has been reported from an LSPR sensor^54^, albeit for an atomic-layer deposited Al_2_O_3_ film.

However, it should be noted that the dielectric-based resonators^45^ tend to have far higher FOM, with values as high as 30, than LSPR-based resonators^48^, which typically do not exceed 0.3. This is mainly due to their high quality factors that can reach values in excess of 10^6^, resulting in an extremely narrow FWHM^55^. However, measuring such a narrow resonance can be non-trivial, and the factors other than FOM also affect the overall detection resolution of a biosensing system. Indeed, there have been several reports of achieving peak resolutions that are much finer than the FWHM of the resonance peaks^43^. For realistic biosensing applications, the gap size should be optimized for each purpose as well. As shown Fig. 4b, a narrow gap provides a high surface sensitivity, but at the cost of limited operation range. Furthermore, as Fig. 4f shows, too narrow of a gap hinder sensing of large analytes such as proteins; based on deviation of experimental data from the calculated data, we estimate that the gap size should be at least 15 nm if we wish to achieve a compact formation of a BSA layer.

It is also worth of note that the fabrication of arrays of plasmonic ring resonators with well-controlled 5-nm air-gap was realized in large-scale on a 4-inch wafer. Furthermore, given the illumination beam spot size of ~500 μm and the pitch of 800 nm, the measured transmission spectrum represents a sum of more than ~10^5^ resonator spectra, but with limited inhomogeneous broadening. The fact that we can still measure a transmission peak with an apparent Q-factor of 7, which is in agreement with the simulation results for perfectly uniform resonators, indicates that the nanoimprinted disks are sufficiently uniform that we do not need to resort to extreme measures to obtain single-resonator spectra, which greatly facilitates use of the resonators for practical applications.

## Methods

### Fabrication of the resonator arrays

A SiN_x_ /SiO_2_ /SiN_x_ multilayer film was deposited on a 4 inch diameter quartz substrate using an RF sputtering method. A schematic description of the process is shown in Fig. 1a. The SiN_x_ layer thickness was kept constant at 70 nm, while the SiO_2_ layer thickness was varied to be between 40 nm and 75 nm. After the multilayer deposition, a 4-inch wafer sized, hexagonal array of resin pillars with 200 nm diameter and 800 nm pitch was fabricated on the multilayer film using a nanoimprinting method with a UV-curable nanoimprinting resist (mr-UVCur06, micro resist technology GmbH)^41^. The scanning electron microscope (SEM) image of the imprinted pillars array is shown in Fig. 1b. Afterward, the top SiN_x_ layer was etched with an ICP-RIE method to form SiN_x_ disks, using the nano-imprinted pillars as the etch-mask. The pillars were then removed by O_2_ plasma ashing, and the spacer SiO_2_ layer was partially etched using Buffered Oxide Etchant to undercut the top SiN_x_ disks and to form a slot, utilizing the bottom SiN_x_ layer as the etch-stop to protect the quartz substrate. Finally, an Au layer with a Cr adhesion layer was sputter-deposited. The thickness of the Au layer was varied between 45 nm and 90 nm.

### FDTD simulations

To simulate the ring resonator array structure, periodic boundary condition is used for all simulations. The , collision frequency of Au used for simulations were 40 THz. Based on the TEM images shown in Fig. 1d–f, we modelled the resonator shapes as a function of the metal thickness and spacer layer thickness. The bottom metal tip on the sidewall of the disk is assumed to be rounded, and the basal metal layer is assumed to penetrate into the slot as a simple straight sloped line. The thickness of the metal tip on the sidewall, the slope and start of the slope of the bottom metal were obtained by fitting a second-order polynomial to the metal thickness and spacer layer thickness obtained from the experimentally obtained TEM images of actually fabricated samples.

### Transmission measurements

The transmission measurement setup is described in Supplementary Figure S3. All the lenses, polarizers, the sample, and the detector were aligned in a straight line. The incident angle was 90°, normal to the sample surface. Due to the symmetry of the resonator array and the normal angle of incidence, the transmission spectra were expected, and confirmed to be, invariant to the polarization (Data not shown).

### Procedures of the Biosensing experiment

To investigate the effect of a self-assembled BSA monolayer on the resonance spectra, the resonators arrays samples were treated with following process.

1. Cleaning
  - 1.1 Rinsed with Acetone.
  - 1.2 Rinsed with Methanol.
  - 1.3 Rinsed with DI water.
  - 1.4 Blow dried with nitrogen.
2. Resonance spectra as ‘air’ were measured.
3. BSA coating.
  - 3.1 Dipped in the solution of BSA in PBS with concentration of 10%.
  - 3.2 Stayed 3 hours at room temperature, while being continuously shaken at 120 rpm.
  - 3.3 Rinsed with DI water.
  - 3.4 Blow dried with nitrogen.
4. Resonance spectra as ‘with BSA’ were measured.

## Additional Information

**How to cite this article**: Lee, J. *et al*. Ultra sub-wavelength surface plasmon confinement using air-gap, sub-wavelength ring resonator arrays. *Sci. Rep*. **6**, 22305; doi: 10.1038/srep22305 (2016).

## Supplementary Material

Supplementary Information

Supplementary Movie S1

Supplementary Movie S2
