# Supplementary material for: Ultra sub-wavelength surface plasmon confinement using air-gap, sub-wavelength ring resonator arrays
Source: Sci Rep. 2016 Feb 29;6:22305. doi: 10.1038/srep22305 (PMC4770440; doi:10.1038/srep22305)
Supplement: Supplementary Information [file srep22305-s1.pdf]

Supplementary Information

Ultra sub-wavelength surface plasmon confinement using air-gap, sub-wavelength ring resonator arrays.

Jaehak Lee[1], Sangkeun Sung[2], Jun-Hyuk Choi[2], Seok Chan Eom[1],

N. Asger Mortensen[3] and Jung H. Shin[4,1]★

★*jhs@kaist.ac.kr*

[1]*Department of Physics, KAIST 373-1 Guseong-dong, Yuseong-Gu, Daejeon, South Korea*

[2] *Korea Institute of Machinery & Materials, 156 Gajeongbuk-Ro, Yuseong-Gu, Daejeon 305-343, South Korea*

[3]*Department of Photonics Engineering, Technical University of Denmark, DK-2800 Kongens Lyngby, Denmark*

[4]*Graduate School of Nanoscience and Technology, KAIST 373-1 Guseong-dong, Yuseong-Gu, Daejeon, South Korea*

Movie S1 (a) and (b) (http://www.nature.com/scientificreports) show propagations of resonant and non-resonant light, respectively, normally incident on a 5-nm gap resonator. At resonance, the incident wave is funneled through the gap so that a large fraction of the incident light is transmitted. Off resonance, the incident wave is nearly completely reflected, with very little transmission through the resonator.

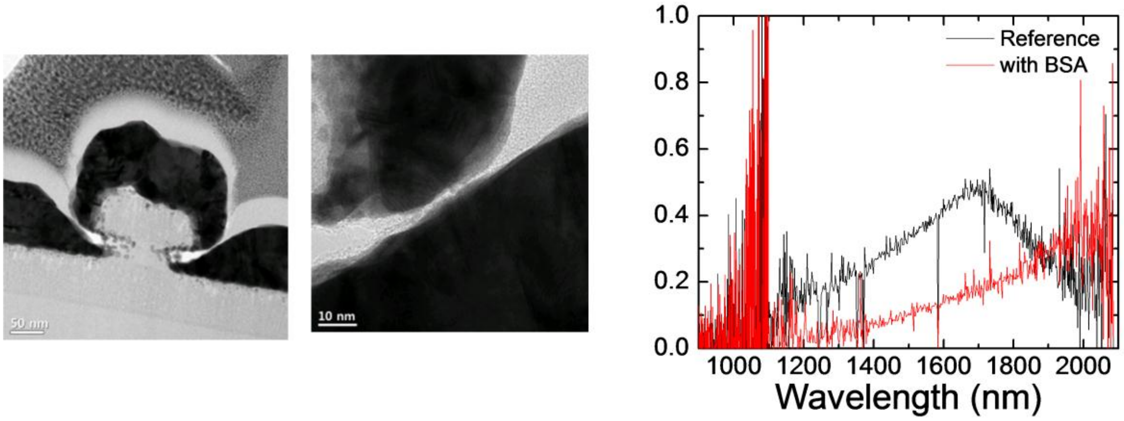

Figure S2. Cross-sectional TEM image and measured transmission spectrum of 3 nm gap resonator sample

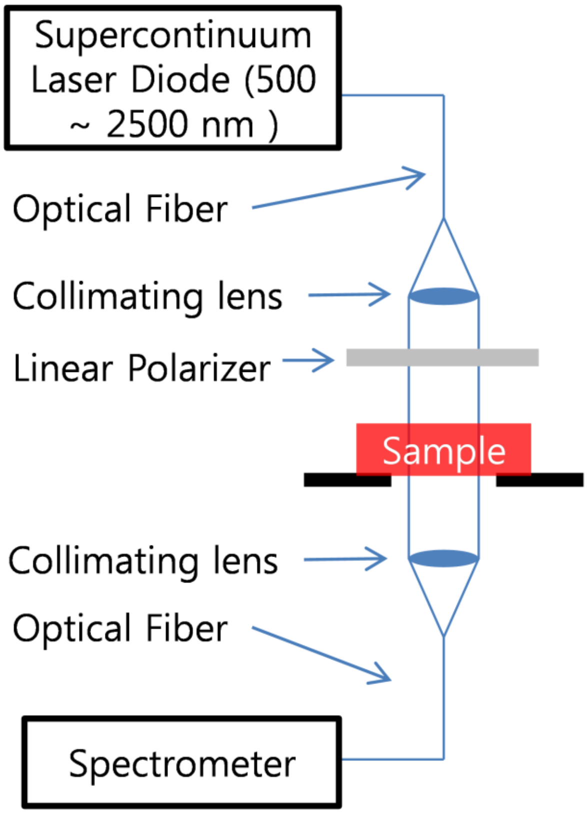

Figure S3. Schematic of the transmission measurement setup.
